# Associations between prenatal exposure to cadmium and lead with neural tube defect risks are modified by single-nucleotide polymorphisms of fetal *MTHFR* and *SOD2*: a case–control study

**DOI:** 10.1186/s12940-021-00752-9

**Published:** 2021-06-05

**Authors:** Mengyuan Liu, Jinhui Yu, Zaiming Su, Ying Sun, Yaqiong Liu, Qing Xie, Zhiwen Li, Linlin Wang, Jie Zhang, Lei Jin, Aiguo Ren

**Affiliations:** 1 Institute of Reproductive and Child Health/Key Laboratory of Reproductive Health, National Health Commission of the People’s Republic of China, Peking University, Beijing, China; 2 Department of Epidemiology and Biostatistics, School of Public Health, Peking University, Beijing, China; 3 Beijing Obstetrics and Gynecology Hospital, Capital Medical University, Beijing, China

**Keywords:** Cadmium, Lead, Neural tube defects, Single-nucleotide polymorphisms, Superoxide dismutase 2 (SOD2), 5,10-methylenetetrahydrofolatereductase (MTHFR)

## Abstract

**Background:**

Prenatal exposure to heavy metals is implicated in the etiology of birth defects. We investigated whether concentrations of cadmium (Cd) and lead (Pb) in umbilical cord tissue are associated with risk for neural tube defects (NTDs) and whether selected genetic variants of the fetus modify their associations.

**Methods:**

This study included 166 cases of NTD fetuses/newborns and 166 newborns without congenital malformations. Umbilical cord tissue was collected at birth or elective pregnancy termination. Cd and Pb concentrations were assessed by inductively coupled plasma-mass spectrometry, and 20 single-nucleotide polymorphisms (SNPs) in 9 genes were genotyped. Odds ratios (ORs) with 95% confidence intervals (CIs) were used to estimate the risk for NTDs in association with metal concentrations or genotype using logistic regression. Multiplicative-scale interactions between the metals and genotypes on NTD risk were assessed with logistic regression, and additive-scale interactions were estimated with a non-linear mixed effects model.

**Results:**

Higher concentrations of Cd were observed in the NTD group than in the control group, but no difference was found for Pb. Concentrations of Cd above the median level showed a risk effect, while the association between Pb and NTD risk was not significant in univariate analyses. The association of Cd was attenuated after adjusting for periconceptional folic acid supplementation. Fetuses with the AG and GG genotypes of rs4880 in *SOD2* (superoxide dismutase 2) tended to have a lower risk, but fetuses with the CT and TT genotypes of rs1801133 in *MTHFR* (5,10-methylenetetrahydrofolatereductase) have a higher risk for NTDs when compared to their respective wild-type. rs4880 and Cd exhibited a multiplicative-scale interaction on NTD risk: the association between higher Cd and the risk for NTDs was increased by over fourfold in fetuses carrying the G allele [OR 4.43 (1.30–15.07)] compared to fetuses with the wild-type genotype. rs1801133 and Cd exposure showed an additive interaction, with a significant relative excess risk of interaction [RERI 0.64 (0.02–1.25)].

**Conclusions:**

Prenatal exposure to Cd may be a risk factor for NTDs, and the risk effect may be enhanced in fetuses who carry the G allele of rs4880 in *SOD2* and T allele of rs1801133 in *MTHFR*.

**Supplementary Information:**

The online version contains supplementary material available at 10.1186/s12940-021-00752-9.

## Background

Neural tube defects (NTDs) are among the most common severe birth defects that include a spectrum of structural malformations caused by failed neural tube closure during early fetal development. The prevalence of this disease remains high in some parts of the world, ranging from 1.3 to 28 in 2000 established pregnancies [1–3]. NTDs are major public health issues due to their high mortality and detrimental impacts on the entire lives of the affected children and their families. Maternal folate deficiency is associated with elevated risk for fetal NTDs [4], and supplementation with folic acid during the periconceptional period can effectively reduce the first occurrence and re-occurrence of the defects [5–8]. However, the mechanisms underlying the association between folate and NTDs remain elusive.

In addition to folate deficiency, other environmental factors may also play a role in NTDs. In populations in which folic acid fortification is implemented, known risk factors, including lack of folic acid supplementation, account for less than half of NTD cases, indicating the need for continued research to identify genetic and environmental factors [9]. Among the variety of environmental factors, heavy metal exposure has attracted much attention. Animal studies have shown that intraperitoneal injection of CdCl2 on gestational day (GD) 8 could induce NTDs in fetal mice [10]. Cadmium (Cd) and lead (Pb) have also exhibited a negative impact on neural tube development in chicks [11, 12] and zebrafish [13]. A few epidemiological studies have investigated the associations between prenatal exposure to Cd or Pb and birth defects. A study conducted in England revealed that a higher concentration of Pb (defined as ≥ 10 μg/L) in domestic drinking water was associated with a greater risk for NTDs [14]. NTD cases had higher levels of Cd and Pb in fetal or maternal serum [15, 16] and in amniotic fluid [17] than controls. In addition, maternal blood Cd and Pb levels were found to be linked to other adverse birth outcomes, such as lower birthweight [18–20]. However, in these studies, prenatal exposure to heavy metals was predominantly assessed in maternal serum or in the external environment, which could only be regarded as a surrogate of fetal exposure, and only a few studies used direct exposure markers from the fetus, which used metal concentrations in umbilical cord blood [16, 19]. In this study, we used umbilical cord tissue to measure metal exposure of the fetus because this tissue has the same origin as the fetus and can exclude potential variations that might be introduced by different placental functions.

In addition, genetics may modify the effect of environmental factors on human health. The toxicity of Cd and Pb is largely due to their ability to destruct the antioxidant system and induce an excessive accumulation of reactive oxygen species (ROS) in tissue and cellular components [21, 22]. Thus, genes involved in the antioxidant pathway may influence the association between heavy metal exposure and NTD occurrence. Selenoproteins, which possess a selenocysteine center, play critical roles in eliminating ROS caused by heavy metals through their antioxidant capabilities [23]. The expression of selenocysteine is regulated by cis-acting elements (i.e., selenocysteine insertion sequence, SECIS) and trans-acting factors, including SECIS binding protein 2 (SBP2), eukaryotic elongation factor selenocysteine-tRNA specific (eEFSec), and ribosomal protein L30 (RPL30). Meanwhile, selenoprotein releases selenium into the central nervous system through apolipoprotein E receptor 2 (ApoER2, encoded by gene LRP8) on the blood–brain barrier, and the transportation of selenoprotein in the nervous system also depends on ApoER2 [24]. Another important mechanism to protect the body from ROS is the superoxide dismutase (SOD) enzymes. SODs catalyze the transformation of superoxide anion (O2• −) into H_2_O_2_ and oxygen; otherwise, superoxide anion would give rise to the generation of ROS [25]. In addition, 5,10-methylenetetrahydrofolatereductase (MTHFR) and methyltetrahydrofolate-homocysteine methyltransferase reductase (MTRR) are crucial enzymes involved in folate one-carbon metabolism, which plays an essential role in neural tube closure through mechanisms yet to be elucidated.

In this study, we investigated the associations between prenatal exposure to Cd and Pb, using their concentrations in umbilical cord tissue, and the risk for NTDs, and examined possible interactions between Cd and Pb exposure and 20 SNPs in 9 genes involved in detoxification and folate metabolism pathways on the occurrence of NTDs.

## Materials and methods

### Study subjects

This case–control study was conducted with subjects recruited from Shanxi Province of northern China [26]. This province is characterized by heavy coal mining and coal-consuming industries, mountainous and arid land, and a high prevalence rate of NTDs, 31.5/10,000 in 2014 [27]. The subjects included fetuses from elective termination of pregnancy as well as newborn infants. NTD cases were diagnosed by fetal ultrasound scan or physical examination at birth or pregnancy termination. 1:1 matched healthy newborns were recruited from the same hospital according to maternal residence (same county) and the date of last menstruation (± 4 weeks with the case mother). Overall, this study included 166 NTD cases (51 with anencephaly, 98 with spina bifida, and 17 with encephalocele), and 166 non-malformed controls recruited during 2004 and 2016. All the subjects included in this study were Han Chinese. This study was approved by the biomedical ethics committee of Peking University (Beijing, China), and all mothers provided consent before participation.

### Questionnaire interview

A structured questionnaire was administered by local health-care professionals through face-to-face interviews with the mothers before discharge from the birthing hospitals. The questionnaire included sociodemographic information, date of last menstruation, disease history, history of pregnancy affected by birth defects, periconceptional folate fortification, and factors related to environmental exposure of toxicity and dietary habits.

### Laboratory assessment of Cd and Pb concentrations

Umbilical cord tissues on the fetal end were collected immediately after normal delivery or pregnancy termination and subsequently stored at -20 °C in a polyethylene bag. When element assessment was performed, approximately 2 g of umbilical cord tissue samples were taken. First, the samples were thawed at 4 °C, rinsed 3 times with deionized water, and blotted on absorbent paper to remove excess water. Next, the tissues were cut into pieces for assessment. Each sample was weighed and put into a glass jar, which was subsequently covered by ventilated aluminum foil and freeze-dried in a CHRIST freeze dryer (ALPHA 2–4 LSC, Martin Christ Gefriertrocknungsanlagen GmbH, Osterode am Harz, Germany). The jar was set on the rack of the solidifying dryer, which was next put in a refrigerator at -70 °C for at least 4 h. The vacuum pump was preheated for 30 min, and then the rack was moved into the drying chamber.

For each assessment, 0.2 g of dried umbilical cord specimen was accurately weighed and digested in a solution of 3 mL of nitric acid (BV-grade III) and 0.5 mL of hydrogen peroxide in a high-pressure microwave acid digestion system (Ultra WAVE, Milestone, Italy) with quart vessels for 1 h. After digestion, 1 mL of the sample was moved to a 2.0 mL Eppendorf tube, and 0.1 mL of the internal standard agent (rhodium concentration as 20 ng/mL), and super-pure deionized water (18.2 MΩ.cm) was added to reach a final volume of 2 mL. This solution was finally used to determine Cd and Pb concentrations with inductively coupled plasma mass spectrometry (ICP-MS) [28] (Agilent Technologies, 7700x, Hachioji, Japan).

The standard curves were calibrated and validated by certified standards of Chinese national reference material (GSB 04–1714 – 2004). As mentioned above, the ICP-MS system was calibrated using rhodium as the internal reference. To eliminate the effects of possible contamination during the process of digestion and sample preparation, a blank solution was prepared and carried through every 20 samples analyzed. The correlation coefficients of all calibration lines were > 0.999. The reference sample, which was made of pig liver (GBW10051), was carried through each lot of samples analyzed to test the stability of the process. The limits of detection (LOD) for Cd and Pb were 0.006 ng/ mL and 0.0172 ng/ ml, respectively. The detection rate for Cd and Pb were 98.2 and 100%, respectively.

### SNP selection

As discussed in the background, we focused on genes that are involved in detoxification and folate metabolism pathways. The SNPs included in this study were either those that have been reported in the literature or the tagSNPs of the analyzed gene, which were obtained by Haploview4.2 software. In total, 20 SNPs were selected (Supplementary Table 1): *SEPP1* (rs7579, rs230820); *SBP2* (rs74458996, rs76367332, rs3211707, rs80298072); *SEP15* (rs5859); eEFSec (rs10934853, rs2977565, rs77776385, rs1702118, rs2981017); *RPL30* (rs150471706, rs4735522); *LRP8* (rs3737983, rs2297660); *SOD2* (rs4880, rs5746105); *MTRR* (rs3776467); *MTHFR* (rs1801133). The call rates for genotyping of all SNPs were higher than 98.8%

### DNA extraction and SNP genotyping

Genomic DNA was extracted from umbilical cord tissue according to the manufacturer’s instruction (blood or tissue genomic DNA extraction kit, TIANGEN, Beijing). The DNA purity was measured by the Nanodrop microspectrophotometer (Thermo Fisher Scientific. Inc), and the A260/A280 ratio was controlled within 1.8–2.0. The DNA sample was then stored in at -20 °C until genotyping.

The DNA template containing the SNPs locus region was amplified by PCR technology, and then the PCR product was subjected to a single base extension reaction using specific extension primers. High-throughput genotyping of single nucleotide polymorphisms (SNPs) was performed using Matrix-Assisted Laser Desorption Ionization Time of Flight Mass Spectrometry (MALDI-TOF–MS) in a gene mass spectrometry system (Sequenom, San Diego, CA, USA).

### Statistical analysis

The distribution of Cd and Pb concentrations was illustrated as median together with interquartile interval (P25–P75). The concentrations of Cd and Pb were compared between the NTD cases and controls through nonparametric analysis. Cd and Pb concentrations were dichotomized into two levels by the median of all subjects. Associations between Cd and Pb exposure and risks for NTDs were evaluated by odds ratio (OR) through logistic regression, and the precision of the estimation was indicated by its 95% confidence interval (95% CI). Periconceptional folate supplementation was included in the model as a confounder in multivariable logistic regression analysis. Since dichotomized variables are easily understandable and interpretable, especially in analyzing two-factor interactions, we categorized Cd and Pb concentrations by their median concentrations.

Hardy–Weinberg Equilibrium (HWE) was tested for each SNP, and if the genotype distribution did not conform with HWE, the SNP would not be included in subsequent analysis. The associations between SNPs and the risk for NTDs were evaluated by unconditional logistic regression with or without the adjustment of folic acid supplementation. Next, we stratified our logistic regression model according to genotypes and produced a cross-product term (multiplicative scale) to evaluate the potential modifying effect of a specific genotype on the association between Cd or Pb levels and NTD risk. Meanwhile, we calculated additive scale interaction using the non-linear mixed effects model described by Demidenko [29]. Statistical analyses were performed with SPSS Version 26.0. A two-sided *P* value of < 0.05 was considered statistically significant.

We chose dichotomized model to analyze the data of this study, although continuous variables may be better powered. However, if the relationship to be examined is nonlinear, the use of continuous variables may miss the opportunity to find such a relationship. In addition, dichotomized variables are directly understandable and interpretable, especially for two-factor interaction analyses.

## Results

Maternal demographic and obstetric characteristics of NTD cases and controls are illustrated in Table 1. The case group had a shorter gestation and a higher pre-pregnancy body mass index (BMI), were more likely to report a history of pregnancy affected by birth defects and fever or flu in the first trimester but less likely to take a folic acid supplement. No differences in maternal age, parity, maternal occupation, and smoking or passive smoking were observed between the two groups.

**Table 1:** Maternal characteristics of neural tube defect cases and controls

Characteristic	Cases (*n* = 166) ^a^ No. (%)	Control (*n* = 166) ^a^ No. (%)	*P* ^b^
Maternal age (years)			0.259
< 25	57 (35.2)	63 (38.4)	
25–29	46 (28.4)	55 (33.5)	
≥ 30	59 (36.4)	46 (28.0)	
High school or above education	27 (16.6)	54 (32.5)	0.001
Maternal farming occupation	129 (77.7)	126 (75.9)	0.795
Pre-pregnancy body mass index ≥ 24	86 (53.4)	61 (37.7)	0.005
Maternal smoking or second-hand smoking	134 (85.4)	131 (80.4)	0.300
Maternal folic acid use	66 (40.5)	81 (52.9)	0.032
Parity			0.424
Primiparae	89 (56.0)	84 (51.5)	
Multiparae	70 (44.0)	79 (48.5)	
Gestational weeks at sample collection			< 0.001
< 37	138(83.1)	6(3.6)	
≥ 37	28(16.9)	160(96.4)	
History of pregnancy affected by birth defects	9 (5.5)	1 (0.6)	0.020
Maternal fever or flu in 1st trimester	59 (36.6)	25 (15.3)	< 0.001

^a^The sum of the numbers may not equal the total number due to missing or unknown data

^b^Comparison of the distribution of each characteristic between NTD cases and controls with the chi-squared test or Fisher’s exact test

Medians with interquartile intervals of Cd and Pb concentrations in the two groups are presented in Table 2. The median concentration of Cd was significantly higher in cases (1.30 ng/g) than in controls (0.93 ng/g) by non-parametric test. No significant difference in Pb concentrations was observed between the two groups.

**Table 2:** Median (P25–P75) concentration of cadmium (Cd) and lead (Pb) assessed in cord tissue (dry weight) in neural tube defect cases and controls

Metal	All subjects	Cases	Controls	*P* ^a^	Maximum–minimum
Cd (ng/g)	1.10 (0.48–3.09)	1.30 (0.57–3.49)	0.93 (0.44–2.68)	0.026	23.411–0.034
Pb (ng/g)	26.18 (17.97–48.58)	27.32 (18.37–50.34)	24.62 (17.17–46.86)	0.211	225.572–4.330

*Abbreviations*: *P25* 25th percentile, *P75* 75th percentile

^a^Comparison of median values between two groups conducted using the Mann–Whitney U test

Table 3 presents the associations between levels of Cd and Pb and the risk for NTDs. The association between Cd levels and NTD risks was statistically significant in univariate logistic regressions [OR, 1.55 (1.00–2.38), *P* = 0.049], whereas the Pb concentrations were not evident [OR, 1.27 (0.83–1.96), *P* = 0.273]. Since folic acid supplementation was associated with lower concentrations of Cd and Pb in umbilical cord tissue (Supplementary Table 2), folic acid supplementation was adjusted for in multivariable logistic regression. The adjustment for folic acid supplementation attenuated the association between Cd and NTD.

**Table 3:** Logistic regression analysis of cadmium (Cd) and lead (Pb) concentrations and the risk for neural tube defects

Metal level ^a^	Controls [n (%)]	Cases [n (%)]	cOR (95% CI)	*P*	aOR (95% CI)	*P* ^b^
**Cd**						
Low (< 26.18 ng/g)	92 (55.4)	74 (44.6)	1		1	
High (≥ 26.18 ng/g)	74 (44.6)	92 (55.4)	1.55 (1.00–2.38)	0.049	1.44 (0.91–2.28)	0.116
**Pb**						
Low (< 1.10 ng/g)	88 (53.0)	78 (47.0)	1		1	
High (≥ 1.10 ng/g)	78 (47.0)	88 (53.0)	1.27 (0.83–1.96)	0.273	1.23 (0.78–1.94)	0.378

*Abbreviations cOR* crude odds ratio, *aOR* adjusted odds ratio

^a^Dichotomized by the median of all subjects

^b^Adjusted for folic acid supplementation

Associations between genotypes of the 20 SNPs in 9 genes and NTD risk were examined. All the SNPs conformed with HWE except rs5746105 in *SOD2*, which was excluded from subsequent analyses. NTD risk decreased with the number of G allele in *SOD2* rs4880 genotype (*P* = 0.045), while the heterozygous genotype (AG) and homozygous genotype (GG) showed insignificant associations compared with the wild-type [OR, 0.69 (0.40–1.20) for AG, OR, 0.17 (0.02–1.45) for GG] (Table 4). In addition, we observed an increasing trend in NTD risk with the number of T alleles in rs1801133 of *MTHFR* [OR, 1.33 (0.67–2.65) for CT, OR, 1.92 (0.93–3.98) for TT]. Associations between other SNPs and NTD risk are presented in Supplementary Table S3.

**Table 4:** Association between single-nucleotide polymorphisms (SNPs) in fetal *SOD2* and *MTHFR* and risk for neural tube defects

SNP and genotype	Controls [n (%)]	Cases [n (%)]	OR (95% CI)	*P*
***SOD2* rs4880**				
AA	104 (72.2)	125 (80.6)	1	
AG	35 (24.3)	29 (18.7)	0.69 (0.40–1.20)	0.190
GG	5 (3.5)	1 (0.6)	0.17 (0.02–1.45)	0.104
*P* for trend				0.045
***MTHFR* rs1801133**				
CC	24 (16.7)	18 (11.6)	1	
CT	78 (54.5)	78 (50.3)	1.33 (0.67–2.65)	0.412
TT	41 (28.7)	59 (38.1)	1.92 (0.93–3.98)	0.080
*P* for trend				0.059

We next examined the interactions between the two SNPs presented in Table 4 and metal levels on NTD risk (Table 5). Rs4880 G allele carriers in *SOD2* (AG and GG genotypes) who had high Cd exposure exhibited a much stronger association with NTD risk, over fivefold higher, compared to fetuses who had the same genotype but had low Cd exposure [OR, 5.48 (1.90–15.82), *P* = 0.002]. No such modification effect for the AA genotype was present. Multiplicative-scale interaction between this SNP and Cd exposure was statistically significant, with an OR of 4.83 (1.48–15.76). The interactive effects were still significant after adjustment for periconceptional folic acid supplementation.

**Table 5:** Interaction between single-nucleotide polymorphisms (SNPs) in fetal *SOD2* and *MTHFR* and cadmium (Cd) exposure on NTD risk

SNP and genotype	Cd level	Cases	Controls	cOR (95% CI)	*P*	aOR (95% CI)	*P* ^a^
***SOD2* rs4880**							
AA	Low	61	54	1		1	
	High	64	50	1.13 (0.67–1.91)	0.638	1.08 (0.63–1.87)	0.776
GG&AG	Low	7	25	1		1	
	High	23	15	5.48 (1.90–15.82)	0.002	3.77 (1.15–12.36)	0.028
Multiplicative interaction ^b^				4.83 (1.48–15.76)	0.009	4.43 (1.30–15.07)	0.017
Additive interaction ^c^				0.77 (0.46–1.09)	< 0.0001	0.76 (0.42–1.11)	< 0.0001
***MTHFR* rs1801133**							
CC	Low	11	11	1			
	High	7	13	0.54 (0.16–1.87)	0.329	0.87 (0.23–3.32)	0.834
CT & TT	Low	57	68	1			
	High	80	51	1.87 (1.14–3.08)	0.014	1.55 (0.91–2.64)	0.106
Multiplicative interaction ^b^				3.48 (0.91–13.25)	0.068	2.68 (0.67–10.74)	0.164
Additive interaction ^c^				0.74 (0.27–1.20)	0.0019	0.64 (0.02–1.25)	0.042

*Abbreviations*: *cOR* crude odds ratio, *aOR* adjusted odds ratio

^a^Adjusted for folic acid supplementation

^b^Multiplicative interaction was measured by a cross-product term by genotype and metal level

^c^Additive interaction was measured by the relative excess risk of interaction (RERI)

For *MTHFR* rs1801133, T allele carriers (CT and TT genotypes) with a higher Cd exposure level showed an increased risk for NTDs [OR, 1.87 (1.14–3.08)], while no such modification effect was found for fetuses with the wildtype (CC). This association was attenuated when folic acid supplementation was adjusted for. No multiplicative-scale interaction between this SNP and Cd exposure on NTD risk was observed, but an additive-scale interaction was found with a relative excess risk of interaction of 0.64 (0.02–1.25) after adjustment for folic acid supplementation.

## Discussion

In this case–control study, we investigated the associations between concentrations of Cd and Pb in umbilical cord tissue and 20 SNPs in 9 genes in metal or folate metabolic pathways with NTD risk, as well as the interactions between metals and SNPs. A suggestive association between Cd levels and NTD risk was observed, whereas the association between Pb level and NTD risk was not evident. More importantly, the SNP rs4880 in *SOD2* (AG & GG vs. AA) and higher Cd exposure showed a multiplicative-scale interaction on NTD risk, and the SNP rs1801133 in *MTHFR* (CT and TT vs. CC) and higher Cd exposure exhibited an additive-scale interaction on NTD risk, supporting an environmental-genetic interaction in the etiology of NTDs.

Data on Cd and Pb concentrations in umbilical cord tissue are scarce. In a Japanese study [30], which used the same methods of metal assessment as our present study, the median values and interquartile ranges of concentrations of Cd and Pb in umbilical cord from healthy newborn were 1.16 (0.87–1.68) ng/g for Cd, and 39.9 (33.8–50.1) ng/g for Pb. The Cd level was similar to that of the control group of our present study [Cd, 0.93 (0.44–2.68) ng/g], while the Pb level is higher than our result [Pb, 24.62 (17.17–46.86) ng/g]. On the other hand, several studies have determined concentrations of these two metals in umbilical cord blood [19, 31, 32]. In a Turkish study, the levels of Cd and Pb were both significantly higher in NTD cases than in control subjects, as detected either in maternal or infant plasma [16]. So far, we did not find any previous research that explored the relationship between Cd or Pb in cord tissue and the risk for NTDs.

We found that maternal fever in the 1st trimester was associated with NTD risk. Hyperthermia during pregnancy has been reported to have a teratogenic effect [33], particularly on the central nervous system [34, 35]. Moreover, the teratogenicity of hyperthermia is closely related to the timing of embryogenesis. Fever in the 1st trimester has been associated with brain damage and NTDs [35], while fever during the 2nd trimester has been linked to autism [36] and abnormalities of psychological development [37]. Animal studies further validated the relationship between hyperthermia in early pregnancy and NTDs [38].

Rs4880 is the most extensively studied SNP in *SOD2* (MnSOD). This variant causes the alteration of valine amino acid (GTT) to alanine (GCT) at codon 16 (Ala16Val), which leads to a structural mutation in the mitochondrial targeting sequence (MTS) [39]. MTS is critical for MnSOD to be recognized and enter the mitochondria to become active and exert its function. The Ala-MnSOD variant exhibits a higher transport efficiency into the mitochondria than Val-MnSOD, which might be due to the transition from an alpha-helix structure (Ala-MnSOD) to a beta-sheet structure (Val-MnSOD) [40]. It has been verified in vitro that MnSOD activity is higher in Ala variants than Val variants [40, 41]. Associations between this variant and a variety of diseases have been extensively investigated; however, there is still no consistent result for definitive assignment of which variant is the culprit for disease occurrence [42]. According to the Genome Aggregation Database (gnomAD, http://gnomad-sg.org/), the percentages of the G allele (Ala variant) in most ethnicities are around 50%, whereas, in East Asia, the frequency is at 0.149, which is generally consistent with the result of the present study (0.10 in the control group). As far as we know, this is the first study to link this gene variant with NTD risk, which demonstrated lower odds of NTDs in G allele carriers. The protective role of G alleles might be due to intact MnSOD activity, which is otherwise impaired in the AA genotype. In the present study, G allele carriers with high Cd exposure was associated with much higher NTD risk when compared to fetuses who did not carry G alleles, suggesting the modification effect of this SNP on the NTD risk in association with Cd exposure. This result was consistent with another study, in which peripheral blood mononuclear cells (PBMCs) derived from people with GG genotype showed higher sensitivity to pyridostigmine bromide (PB)-induced oxidative stress [43]. On the other hand, another SNP of SOD2 investigated in this study, rs5746105, showed Hardy–Weinberg disequilibrium. This deviation might be due to the limited sample size. The power of the HWE test of this SNP was 0.22, as calculated by PASS version 11.

*MTHFR* (5,10-methylenetetrahydrofolatereductase) encodes a crucial enzyme in folate metabolism, which catalyzes the conversion of 5,10-methylenetetrahydrofolate to 5-methyltetrahydrofolate. C677T (rs1801133) is a common polymorphism in *MTHFR*. This variant hampers the enzyme activity [44] and is consistently linked with elevated plasma total homocysteine levels [45], which has been related to the genesis of NTDs [46, 47]. The prevalence of T alleles is diverse among ethnicities, being approximately 0.29 in the East Asian population according to the gnomAD database. However, the frequency of the T allele in the present study is approximately 0.59 (case group, 0.63; control group, 0.56), which is much higher than this value. A large-scale epidemiological study with over 20,000 Chinese participants revealed a T allele frequency of 0.49 [48]. These results indicated that the Chinese population might possess a higher frequency of T alleles than other populations in Eastern Asia, which might be attributable to a higher NTD risk in China [49]. Either in the mother or the child, T alleles confer a modestly increased NTD risk in the allelic (additive) model [47, 50], but the results were not always consistent among populations [51]. We found that the T allele enhances the risk effect of Cd exposure on NTD risk, suggesting that T allele carriers are more vulnerable to adverse environmental factors, even though the genotypic factor alone did not confer a significant increase in NTD risk. The possible mechanism underlying this interaction may also be related to oxidative stress. Numerous studies have shown that folate has anti-oxidative properties, and homocysteine can induce oxidative stress [52, 53]. Subjects carrying the TT genotype in *MTHFR* rs1801133 have lower folate levels and higher homocysteine levels [44, 47], which may exacerbate the effect of oxidative stress induced by Cd exposure.

The majority of the women included in this study lived in rural areas and reported farming as their occupation. No occupational exposure or facility-associated sources of metals were identified. However, the province from which the study subjects were recruited has rich coal reserves and production [54]. Coal is the main source of energy for domestic heating and cooking, and coal-consuming industries were prevalent in the province. A previous study suggested that coal combustion was responsible for the deposition of heavy metals in the air in the Shanxi basin [55]. Contents of Cd and Pb in coal produced in Shanxi Province are higher than those from the United States [56]. These findings suggest that residents in Shanxi Province may live with an aggravated environmental burden of Cd and Pb, which might be partly responsible for the higher NTD risk in the population.

Several strengths and limitations of this study should be addressed. First, the umbilical cord tissue has been scarcely investigated as an environmental exposure indicator for fetuses. As a tissue that shares a common origin with the fetus body and develops in the same environment, umbilical cord tissue should be a better biospecimen for assessing fetal exposure than placenta tissue, maternal blood, or maternal scalp hair. Metal concentrations in cord tissue may represent long-term exposure of the fetus. Second, we combined genetic and environmental factors (i.e., Cd or Pb exposure levels) to analyze their interrelationships on NTD risk, which provided a more comprehensive picture to understand the etiology of NTDs. However, due to the limited number of subjects, the power to detect the association between metals or genetic variants and NTD risks is limited. Second, although the selection of the SNPs in this study was based on a candidate gene approach, examination of the associations for 20 SNPs may result in false-positive results in a study with a limited sample size like the present one. Thus, the conclusion drawn from this study needs to be replicated in the future with larger sample sizes. Another limitation lies in the timing during which we collected the umbilical cord tissues, which did not parallel the time frame of neural tube closure due to the intrinsic nature of the outcomes examined. Thus, the metal concentration we obtained in this study might not exactly reflect the exposure levels during neural tube closure.

## Conclusion

Higher concentrations of Cd, but not Pb, in umbilical cord tissue were associated with a higher risk for NTDs. G alleles in *SOD2* rs4880 tended to be associated with a reduced risk, but T alleles in *MTHFR* rs1801133 to be associated with an increased risk for NTDs. Moreover, G alleles of rs4880 and T alleles of rs1801133 might enhance the association between higher Cd level and increased NTD risk, as compared to their wild-type genotypes. This study provided novel evidence on gene-environment interactions in the etiology of NTDs, but the findings need to be substantiated in large-scale studies in the future.

## Supplementary Information

**Additional file 1: Supplementary Table S1**. Information of 20 single nucleotide polymorphisms (SNPs) of 9 genes examined in this study. **Supplementary Table S2**. Median (P25–P75) concentration of elements with and without folic acid supplementation. **Supplementary Table S3**. Association between SNP and odds of NTDs with no statistical significance.

## Data Availability

The datasets generated and/or analyzed during the current study are not publicly available due to ethical and legal reasons but are available from the corresponding author on reasonable request.

## Abbreviations

Cd: Cadmium
Pb: Lead
Mn: Manganese
NTDs: Neural tube defects
SNPs: Single nucleotide polymorphisms
OR: Odds ratio
RERI: Relative excess risk of interaction
MTHFR: 5,10-Methylenetetrahydrofolatereductase
SOD: Superoxide dismutase
HWE: Hardy–Weinberg Equilibrium
