# Correction to: Associations between prenatal exposure to cadmium and lead with neural tube defect risks are modified by single-nucleotide polymorphisms of fetal *MTHFR* and *SOD2*: a case–control study

**DOI:** 10.1186/s12940-021-00762-7

**Published:** 2021-07-12

**Authors:** Mengyuan Liu, Jinhui Yu, Zaiming Su, Ying Sun, Yaqiong Liu, Qing Xie, Zhiwen Li, Linlin Wang, Jie Zhang, Lei Jin, Aiguo Ren

**Affiliations:** 1 Institute of Reproductive and Child Health/Key Laboratory of Reproductive Health, National Health Commission of the People’s Republic of China, Peking University, Beijing, China; 2 Department of Epidemiology and Biostatistics, School of Public Health, Peking University, Beijing, China; 3 Beijing Obstetrics and Gynecology Hospital, Capital Medical University, Beijing, China

**Correction to: Environmental Health 20, 66 (2021)**

**https://doi.org/10.1186/s12940-021-00752-9**

Following the publication of the original article [1], the author reported an error in the affiliation list of Dr. Lei Jin and the address for the correspondence. Dr. Jins’ affiliation should only be 1 and 2, and the address for both corresponding authors should be Institute of Reproductive and Child Health/Key Laboratory of Reproductive Health, National Health Commission of the People’s Republic of China, Peking University, Beijing, China.

The affiliation number and the correspondence address are corrected above and the original article has been corrected.
