# Supplementary material for: Associations between prenatal exposure to cadmium and lead with neural tube defect risks are modified by single-nucleotide polymorphisms of fetal MTHFR and SOD2: a case–control study
Source: Environ Health. 2021 Jun 5;20:66. doi: 10.1186/s12940-021-00752-9 (PMC8180011; doi:10.1186/s12940-021-00752-9)
Supplement: Supplementary file 1 — Additional file 1: Supplementary Table S1. Information of 20 single nucleotide polymorphisms (SNPs) of 9 genes examined in this study. Supplementary Table S2. Median (P25–P75) concentration of elements with and without folic acid supplementation. Supplementary Table S3. Association between SNP and odds of NTDs with no statistical significance. [file 12940_2021_752_MOESM1_ESM.docx]

**Supplementary Information**

**High concentrations of aluminum in maternal serum and placental tissue are associated with increased risk for fetal neural tube defects**

Mengyuan Liu^1,2^, Jinhui Yu^1,2^, Zaiming Su^1,2^, Ying Sun^1,2^, Yaqiong Liu^1,2^, Qing Xie^1,2^, Zhiwen Li^1,2^, Linlin Wang^1,2^, Jie Zhang^1,2^, Lei Jin^1,2^*, Aiguo Ren^1,2,3^*

**Affiliation:**

^1^Institute of Reproductive and Child Health/Key Laboratory of Reproductive Health, National Health Commission of the People’s Republic of China, Peking University, Beijing, China

^2^Department of Epidemiology and Biostatistics, School of Public Health, Peking University, Beijing, China

^3^Beijing Obstetrics and Gynecology Hospital, Capital Medical University, Beijing, China

***Corresponding authors**:

Dr. Lei Jin, Institute of Reproductive and Child Health, Peking University Health Science Center, Beijing 100191, China. Phone: +86-1082801759-111, E-mail: jinlei[@bjmu.edu.cn](mailto:jinlei@bjmu.edu.cn)

Prof. Aiguo Ren, Institute of Reproductive and Child Health, Peking University Health Science Center, Beijing 100191, China. Phone: +86-1082801140, E-mail: renag[@bjmu.edu.cn](mailto:jinlei@bjmu.edu.cn)

**Supplementary Table S1.** Information of 20 single nucleotide polymorphisms (SNPs) of 9 genes examined in this study.

| Genes full name | Genes abbreviation | Chromosome position | SNPs | Variation | Location | Effect on protein | Minor allele | Minor allele in Chinese | MAF / total number of subjects in Chinese study | Reference |
| --- | --- | --- | --- | --- | --- | --- | --- | --- | --- | --- |
| Selenoprotein P | *SEPP1/SELENOP* | 5p12 | rs7579 | C>T | 3'-UTR | affects the selenoprotein transport and enzyme activities[1] | T | T | 0.28/1400 | [2] |
|  |  |  | rs230820 | A>C | Intron | - | A | C^a^ | - | - |
| SECIS binding protein 2 | *SBP2* | 9q22.2 | rs74458996 | G>C | Intron | - | C | C^a^ | - | - |
|  |  |  | rs76367332 | G>C | Intron | - | C | C^a^ | - | - |
|  |  |  | rs3211707 | G>C | 2kb upstream | - | C | C^a^ | - | - |
|  |  |  | rs80298072 | T>C | Intron | - | C | C^a^ | - | - |
| Selenoprotein F | *SEP15* | 1p22.3 | rs5859 | C>T | Non-Coding Transcript | reduce the efficiency of selenium incorporation into SEP15[3] | T | T | 0.035/206 | [4] |
| Eukaryotic elongation factor, selenocysteine-tRNA specific, SelB | *eEFSec* | 3q21.3 | rs10934853 | C>A | Intron | - | A | A | 0.43/1370 | [5] |
|  |  |  | rs2977566 | G>A | Intron | - | A | A^a^ | - | - |
|  |  |  | rs77776385 | T>G | Intron | - | G | G^a^ | - | - |
|  |  |  | rs1702118 | T>G | Intron | - | G | G^a^ | - | - |
|  |  |  | rs2981017 | C>T | Intron | - | T | T^a^ | - | - |
| RibosomalproteinL30 | *RPL30* | 8q22.2 | rs150471706 | C>T | 500b Downstream | - | T | T^a^ | - | - |
|  |  |  | rs4735522 | C>T | 2kb Upstream | - | T | T^a^ | - | - |
| Apolipoprotein E receptor2, ApoER2 | *LRP8* | 1p32.3 | rs3737983 | G>A | Exon | Synonymous variant | A | G^a^ | - | - |
|  |  |  | rs2297660 | G>T | Intron | Synonymous variant | T | G^a^ | - | - |
| Superoxide dismutase 2 | *SOD2* | 6q25.3 | rs4880 | A>G | Exon | reduce the activity of SOD2[6] | G | G^a^ | - | - |
|  |  |  | rs5746105 | A>G | Intron | - | G | G^a^ | - | - |
| Methyltetrahydrofolate-homocysteine methyltransferase reductase | *MTRR* | 5p15.31 | rs3776467 | C>T | Intron | - | C | T^a^ | - | - |
| 5,10-methylenetetrahydrofolatereductase | *MTHFR* | 1p36.22 | rs1801133 | C>T | Exon | reduce the activity of MTHFR[7] | T | T | 0.49/20702 | [8] |

^a^Data from gnomAD for East Asia population.

**Supplementary Table S2.** Median (P25–P75) concentration of elements with and without folic acid supplementation.

|  | Folic acid supplementation | Cd (ng/g) | n | Pb (ng/g) | n |
| --- | --- | --- | --- | --- | --- |
| Overall | Yes | 0.82 (0.43-1.57) | 147 | 21.98 (14.15-31.13) | 146 |
|  | No | 1.84 (0.66-4.76) | 168 | 30.57 (20.09-57.33) | 169 |
|  | *P* ^a^ | <0.001 |  | <0.001 |  |
| Cases | Yes | 1.08 (0.46-1.72) | 66 | 24.96 (17.19-38.55) | 66 |
|  | No | 2.03 (0.64-5.05) | 96 | 30.18 (19.54-57.04) | 97 |
|  | *P* ^a^ | 0.004 |  | 0.019 |  |
| Control | Yes | 0.68 (0.35-1.23) | 81 | 19.45 (12.23-29.79) | 80 |
|  | No | 1.58 (0.66-4.67) | 72 | 31.88 (21.09-60.65) | 72 |
|  | *P* ^a^ | <0.001 |  | <0.001 |  |

Abbreviations: Cd, cadmium; Pb, lead; P25, 25th percentile; P75, 75th percentile.

^a^ Comparison of median values between two groups conducted using the Mann-Whitney *U* test.

**Supplementary Table S3.** Association between SNP and odds of NTDs with no statistical significance.

| Factors | Genotype | Controls | Cases | OR (95% CI) | *P* value |
| --- | --- | --- | --- | --- | --- |
| rs7579 | CC | 83 | 80 | 1 |  |
|  | CT | 51 | 62 | 1.26 (0.78-2.04) | 0.344 |
|  | TT | 9 | 11 | 1.27 (0.50-3.22) | 0.104 |
|  | *P* for trend |  |  |  | 0.357 |
| rs230820 | AA | 60 | 52 | 1 |  |
|  | AC | 59 | 77 | 1.51 (0.91-2.49) | 0.111 |
|  | CC | 24 | 22 | 1.06 (0.53-2.10) | 0.873 |
|  | *P* for trend |  |  |  | 0.515 |
| rs74458996 | GG | 101 | 112 | 1 |  |
|  | GC | 41 | 38 | 0.84 (0.50-1.40) | 0.496 |
|  | CC | 3 | 6 | 1.80 (0.44-7.40) | 0.413 |
|  | *P* for trend |  |  |  | 0.952 |
| rs76367332 | GG | 98 | 112 | 1 |  |
|  | GC | 41 | 38 | 0.81 (0.48-1.36) | 0.428 |
|  | CC | 3 | 6 | 1.75 (0.43-7.18) | 0.437 |
|  | *P* for trend |  |  |  | 0.864 |
| rs3211707 | GG | 51 | 55 | 1 |  |
|  | GC | 69 | 76 | 1.02 (0.62-1.69) | 0.934 |
|  | CC | 24 | 25 | 0.97 (0.49-1.90) | 0.920 |
|  | *P* for trend |  |  |  | 0.952 |
| rs80298072 | TT | 91 | 91 | 1 |  |
|  | TC | 46 | 61 | 1.33 (0.82-2.14) | 0.250 |
|  | CC | 8 | 4 | 0.50 (0.15-1.72) | 0.271 |
|  | *P* for trend |  |  |  | 0.832 |
| rs5859 | CC | 134 | 147 | 1 |  |
|  | CT | 9 | 7 | 0.71 (0.26-1.96) | 0.507 |
|  | *P* for trend |  |  |  | - |
| rs10934853 | CC | 50 | 60 | 1 |  |
|  | CA | 69 | 65 | 0.79 (0.47-1.30) | 0.348 |
|  | AA | 24 | 31 | 1.08 (0.56-2.07) | 0.825 |
|  | *P* for trend |  |  |  | 0.961 |
| rs2977565 | GG | 46 | 54 | 1 |  |
|  | GA | 69 | 66 | 0.82 (0.49-1.37) | 0.439 |
|  | AA | 29 | 36 | 1.06 (0.57-1.98) | 0.861 |
|  | *P* for trend |  |  |  | 0.975 |
| rs77776385 | TT | 74 | 78 | 1 |  |
|  | TG | 57 | 63 | 1.05 (0.65-1.69) | 0.846 |
|  | GG | 13 | 15 | 1.10 (0.49-2.46) | 0.826 |
|  | *P* for trend |  |  |  | 0.794 |
| rs1702118 | TT | 109 | 117 | 1 |  |
|  | TG | 31 | 34 | 1.02 (0.59-1.78） | 0.939 |
|  | GG | 5 | 4 | 0.75 (0.20-2.85) | 0.667 |
|  | *P* for trend |  |  |  | 0.841 |
| rs2981017 | CC | 50 | 50 | 1 |  |
|  | CT | 68 | 66 | 0.97 (0.58-1.63) | 0.910 |
|  | TT | 25 | 40 | 1.60 (0.85-3.02) | 0.147 |
|  | *P* for trend |  |  |  | 0.193 |
| rs150471706 | CC | 113 | 118 | 1 |  |
|  | CT | 31 | 36 | 1.11 (0.65-1.92) | 0.702 |
|  | TT | 0 | 2 | - | - |
|  | *P* for trend |  |  |  | 0.420 |
| rs4735522 | CC | 81 | 78 | 1 |  |
|  | CT | 55 | 68 | 1.28 (0.80-2.06) | 0.300 |
|  | TT | 8 | 10 | 1.30 (0.49-3.46) | 0.602 |
|  | *P* for trend |  |  |  | 0.312 |
| rs3737983 | AA | 88 | 82 | 1 |  |
|  | AG | 48 | 64 | 1.07 (0.39-2.91) | 0.900 |
|  | GG | 8 | 10 | 0.75 (0.28-1.98) | 0.556 |
|  | *P* for trend |  |  |  | 0.182 |
| rs2297660 | TT | 65 | 70 | 1 |  |
|  | TG | 66 | 66 | 0.58 (0.26-1.31) | 0.190 |
|  | GG | 11 | 19 | 0.62 (0.28-1.41) | 0.256 |
|  | *P* for trend |  |  |  | 0.502 |
| rs3776467 | CC | 46 | 52 | 1 |  |
|  | CT | 68 | 74 | 0.96 (0.58-1.61) | 0.885 |
|  | TT | 28 | 30 | 0.95 (0.50-1.82) | 0.872 |
|  | *P* for trend |  |  |  | 0.863 |

1. Meplan, C., *Selenium and chronic diseases: a nutritional genomics perspective.* Nutrients, 2015. **7**(5): p. 3621-51.

2. Wu, H., X. Jia, H. Zhao, Y. Huang, C. Liu, Z. Huang, S. Li, and J. Wang, *Identification of SEPP1 polymorphisms is not a genetic risk factor for preeclampsia in Chinese Han women: A clinical trial and experimental study.* Medicine (Baltimore), 2017. **96**(28): p. e7249.

3. Hu, Y.J., K.V. Korotkov, R. Mehta, D.L. Hatfield, C.N. Rotimi, A. Luke, T.E. Prewitt, R.S. Cooper, W. Stock, E.E. Vokes, et al., *Distribution and functional consequences of nucleotide polymorphisms in the 3'-untranslated region of the human Sep15 gene.* Cancer Res, 2001. **61**(5): p. 2307-10.

4. Wu, R., R. Zhang, Y. Xiong, W. Sun, Y. Li, X. Yang, J. Liu, Y. Jiang, H. Guo, X. Mo, et al., *The study on polymorphisms of Sep15 and TrxR2 and the expression of AP-1 signaling pathway in Kashin-Beck disease.* Bone, 2019. **120**: p. 239-245.

5. Liu, F., A.W. Hsing, X. Wang, Q. Shao, J. Qi, Y. Ye, Z. Wang, H. Chen, X. Gao, G. Wang, et al., *Systematic confirmation study of reported prostate cancer risk-associated single nucleotide polymorphisms in Chinese men.* Cancer Sci, 2011. **102**(10): p. 1916-20.

6. Sutton, A., H. Khoury, C. Prip-Buus, C. Cepanec, D. Pessayre, and F. Degoul, *The Ala16Val genetic dimorphism modulates the import of human manganese superoxide dismutase into rat liver mitochondria.* Pharmacogenetics, 2003. **13**(3): p. 145-57.

7. Frosst, P., H.J. Blom, R. Milos, P. Goyette, C.A. Sheppard, R.G. Matthews, G.J. Boers, M. den Heijer, L.A. Kluijtmans, L.P. van den Heuvel, et al., *A candidate genetic risk factor for vascular disease: a common mutation in methylenetetrahydrofolate reductase.* Nat Genet, 1995. **10**(1): p. 111-3.

8. Huo, Y., J. Li, X. Qin, Y. Huang, X. Wang, R.F. Gottesman, G. Tang, B. Wang, D. Chen, M. He, et al., *Efficacy of folic acid therapy in primary prevention of stroke among adults with hypertension in China: the CSPPT randomized clinical trial.* JAMA, 2015. **313**(13): p. 1325-35.

***References***
